# Traditional versus compressive implant osseointegration after immediate placement: A randomized histomorphometric study

**DOI:** 10.6026/973206300220908

**Published:** 2026-02-28

**Authors:** Rajkran Chitumalla, Lalit Narayan Singh, Roshan Samuel, Rashmi R, Afreen Kouser, Meghna Pujara, Tanvi Hirani

**Affiliations:** 1 Department of Restorative and Prosthetic Dental Sciences, College of Dentistry, King Saud bin Abdulaziz University for Health Sciences, King Abdullah International Medical Research Center, Ministry of National Guard-Health affairs, Riyadh, Saudi Arabia; 2 Department of Dentistry, Autonomous State Medical College Sultanpur, Uttar Pradesh, India; 3 Department of Conservative Dentistry and Endodontics, School of Dental Sciences, Krishna Vishwa Vidyapeeth (Deemed to be University) Taluka-Karad, Satara, Maharashtra, India; 4 Department of Prosthodontics, Sri Rajiv Gandhi College of Dental Sciences and Hospital, Bangalore, India; 5 Department of Periodontics, Faculty of Dental Science, Dharmsinh Desai University, Nadiad, Gujarat, India; 6 Department of Periodontology and Implantology, Karnavati School of Dentistry, Karnavati University, Gandhinagar, Gujarat, India

**Keywords:** Immediate implant placement, compressive implant, osseointegration, bone-to-implant contact, primary stability, extraction socket, macrogeometry

## Abstract

Immediate implant placement challenges primary stability and osseointegration in compromised extraction sockets with large gap distances or thin buccal plates. Therefore, it is of interest to compare 40 conventional tapered implants versus 40 compressive-thread implants in 80 patients. Primary outcomes included histomorphometric bone-to-implant contact (BIC%) and bone area fraction occupancy (BAFO%) at 4 months, plus ISQ stability values. Compressive implants showed superior BIC% (68.4 ± 8.7% vs 52.3 ± 9.1%, p<0.001), BAFO% (61.2 ± 7.4% vs 48.9 ± 8.2%, p<0.001), ISQ gains and less buccal resorption (0.41 ± 0.22 vs 1.08 ± 0.39, p<0.001). Compressive macrogeometry advances immediate placement by optimizing early osseointegration and bone preservation in high-risk sites.

## Background:

Immediate implant placement after tooth extraction reduces treatment time, preserves soft tissue architecture and improves patient satisfaction [1]. However, the mismatch between implant geometry and fresh extraction socket morphology often results in large horizontal defects, reduced primary stability and increased risk of buccal bone resorption [2]. Traditional tapered implants rely primarily on apical and palatal/lingual engagement, leaving circumferential gaps that require extensive grafting, especially in the critical buccal corridor [3]. Compressive implants incorporate a progressive thread design with increasing thread depth and width from coronal to apical, combined with a slightly wider body in the coronal third. This macrogeometry is intended to mechanically compress and displace socket bone laterally during insertion, thereby increasing primary stability and bone-to-implant contact even in underprepared sites [4]. Preclinical studies have demonstrated that compressive loading induces osteoblastic activity through controlled micro strain and enhances early bone apposition [5, 6].

Recent animal studies comparing compressive versus conventional implants in immediate placement models have shown superior BIC and bone density at 4-8 weeks with compressive designs [7, 8]. Clinical reports are limited, but preliminary data suggest higher insertion torque and ISQ values with compressive macrogeometry [9]. No randomized controlled trial has yet directly compared histomorphometric osseointegration and long-term marginal bone stability between these two implant designs in human immediate placement protocols. The clinical relevance of improved early osseointegration is substantial. Higher BIC and bone density at early healing stages may reduce micromotion, accelerate secondary stability, minimize crestal bone remodeling, particularly in aesthetic zones where even 0. 5 mm of additional resorption can compromise outcomes [10]. Therefore, it is of interest to compare osseointegration, implant stability evolution, peri-implant bone remodeling between traditional tapered implants and compressive macrogeometry implants in immediate single-tooth replacement. The primary hypothesis was that compressive implants would achieve significantly higher BIC% and BAFO% at 4 months post-placement.

## Materials and Methods:

## Study design and participants:

This prospective, parallel-group, randomized controlled trial was conducted between September 2020 and June 2023 at the Department of Periodontology.

## Inclusion and Exclusion criteria:

## Inclusion criteria:

Age 21-70 years, presence of hopeless tooth with intact socket walls (≥70% of buccal plate), adequate mesiodistal and apicocoronal space, insertion torque ≥25 Ncm.

## Exclusion criteria:

Active infection, buccal plate dehiscence >50%, uncontrolled diabetes (HbA1c >8%), smoking >10 cigarettes/day, bisphosphonate therapy, need for vertical augmentation.

## Randomization and implant systems:

Patients were randomly allocated (1:1) using computer-generated blocks of 10, stratified by tooth type. The Traditional group received Roxolid SLActive tissue-level tapered implants (Bone Level Tapered, Straumann), while the Compressive group received compressive-thread implants with identical surface and material (BLX, Straumann).

## Surgical and prosthetic protocol:

All procedures were performed by one experienced surgeon under local anesthesia. Atraumatic extraction was performed without flap elevation. Osteotomy was initiated with a lance drill followed by sequential shaping using manufacturer-specific drills. In the Traditional group, osteotomy was completed to full diameter; in the Compressive group, underpreparation was performed by stopping one drill size short in soft bone (type III-IV) or two sizes short in dense bone (type I-II) to maximize compression. Implants were placed with the smooth collar 1 mm supra-crestally. Jumping gaps >2 mm were filled with deproteinized bovine bone mineral (Bio-Oss, Geistlich). Healing abutments were placed (Type I immediate restoration not performed). Final screw-retained zirconia crowns were delivered at 16 weeks.

## Outcome measurements:

Primary stability was recorded as insertion torque (Ncm) and ISQ using resonance frequency analysis (Osstell). ISQ was repeated at 2, 6, 16 weeks. At 16 weeks, during prosthetic delivery, a 2. 8 mm trephine biopsy was harvested from the implant shoulder region in the first 25 patients per group (n=50 biopsies) for histomorphometric analysis. Specimens were processed undecalcified, embedded in resin, sectioned longitudinally. BIC% and BAFO% were measured using ImageJ software by a blinded examiner. Marginal bone level changes were measured on standardized periapical radiographs at implant placement, prosthetic delivery, 12 months using parallel technique and ImageJ calibration. Buccal and palatal bone thickness at platform level was measured on CBCT at baseline and 12 months.

## Statistical analysis:

Data were analyzed using SPSS 27. 0. Normality was confirmed with Shapiro-Wilk test. Independent t-tests compared inter-group differences; repeated-measures ANOVA evaluated ISQ changes. Pearson correlation assessed relationships between insertion torques, ISQ, BIC%. Survival was analyzed using Kaplan-Meier. Significance level was p<0. 05.

## Results:

Eighty patients (42 female, 38 male; mean age 48. 7 ± 11. 3 years) were included. Tooth distribution: incisors (52%), canines (18%), premolars (30%). Socket types: II (58%), III (42%). Baseline characteristics showed no significant differences between groups in age, sex, or socket type, though the Compressive group had significantly higher insertion torque (56.8 ± 9.3 vs 38.4 ± 8.7 Ncm, p<0.001) and initial ISQ values (78.9 ± 4.1 vs 71.2 ± 5.4, p<0.001) with comparable buccal gap dimensions (Table 1). At 16 weeks, histomorphometric analysis demonstrated superior osseointegration in the Compressive group, with bone-to-implant contact (BIC%) of 68.4 ± 8.7% vs 52.3 ± 9.1% (p<0.001), bone area fraction occupancy of 61.2 ± 7.4% vs 48.9 ± 8.2% (p<0.001), and reduced marrow space (38.8 ± 7.4% vs 51.1 ± 8.2%) (p<0.001) (Table 2). Qualitative histology confirmed more mature lamellar bone formation with direct implant thread contact and minimal fibrous encapsulation in the Compressive group. ISQ values increased significantly over time in both groups (p<0.001), but the Compressive group maintained consistently higher stability at all timepoints (placement: 78.9±4.1, 2 weeks: 79.4±3.8, 6 weeks: 80.1±3.5, 16 weeks: 79.8±3.2) compared to Traditional (71.2±5.4, 69.8±5.9, 72.1±5.2, 74.1±4.6; all p<0.001), with no secondary stability dip (Table 3). At 12 months post-loading, the Compressive group exhibited significantly less buccal marginal bone loss (0.41 ± 0.22 mm vs 1.08 ± 0.39 mm, p<0.001) and buccal plate thickness reduction (0.38 ± 0.19 mm vs 0.91 ± 0.34 mm, p<0.001), with similar reductions on mesial/distal aspects. Implant survival and success rates were 100% in both groups. Strong correlations existed between insertion torque and final BIC% (r=0.78, p<0.001) and initial ISQ and final ISQ (r=0.81, p<0.001).

## Discussion:

This randomized controlled trial provides the first human histomorphometric evidence that compressive macrogeometry significantly enhances early osseointegration in immediate implant placement. The 30.8% increase in BIC% and 25.2% increase in BAFO% at 16 weeks with compressive implants represent substantial biological advantages over traditional tapered designs. The superior osseointegration can be explained by several biomechanical and biological mechanisms. The compressive thread design generates controlled lateral bone displacement and micro strain in the 1000-3000 µ range, known to stimulate osteoblastic activity and accelerate bone remodeling [11]. Higher insertion torque (56.8 versus 38.4 Ncm) indicates greater bone compression and immediate bone-to-implant apposition, reducing the critical gap that must be bridged by new bone formation [12]. The absence of a secondary stability dip in the Compressive group suggests that mechanical stability was sufficient to prevent fibrous tissue formation during the early healing phase. The significantly reduced buccal bone resorption (0.41 verses 1.08 mm) at 12 months has major clinical implications for aesthetic outcomes. The compressive design appears to provide a buttressing effect on the thin buccal plate through lateral bone displacement, counteracting the natural resorptive remodeling that occurs after extraction [13]. This finding aligns with preclinical studies showing preservation of bundle bone through mechanical loading [14]. These results surpass those reported in previous immediate placement studies using conventional implants, where BIC% typically ranges from 45-55% at 4-6 months [15]. The values achieved with compressive implants approach those observed in healed sites with delayed placement, suggesting that this macrogeometry largely overcomes the biological compromise associated with immediate protocols. The correlation between insertion torques, ISQ, final BIC% validates the use of these clinical parameters as predictors of osseointegration success. The ability to achieve high primary stability without excessive countersinking preserves crestal bone and soft tissue architecture, particularly important in the aesthetic zone [16]. Strengths of this study include the randomized design, standardized surgical protocol, inclusion of histomorphometric analysis, 12-month radiographic follow-up. The flapless approach minimized surgical trauma and preserved blood supply to the buccal plate. Limitations include the relatively short histologic evaluation at 16 weeks and restriction to maxillary anterior/premolar sites. Future studies should evaluate long-term outcomes in molar regions and patients with thinner buccal plates (<1 mm).

## Conclusion:

Compressive macrogeometry implants demonstrate significantly superior early osseointegration compared to traditional tapered implants in immediate placement protocols. These histological advantages translate into higher implant stability throughout healing, absence of secondary stability dip, 62% less buccal marginal bone loss at 12 months. The findings establish compressive implants as a biologically superior option for immediate implantation, potentially allowing more predictable outcomes.

## Figures and Tables

**Table 1:** Baseline patient and surgical parameters

**Parameter**	**Traditional (n=40)**	**Compressive (n=40)**	**p-value**
Age (years)	49.1 ± 11.8	48.3 ± 10.9	0.762
Socket type II/III	23/17	23/17	1
Initial gap buccal (mm)	3.8 ± 1.1	3.9 ± 1.0	0.689
Insertion torque (Ncm)	38.4 ± 8.7	56.8 ± 9.3	<0.001*
Initial ISQ	71.2 ± 5.4	78.9 ± 4.1	<0.001*
*Statistically significant

**Table 2:** Histomorphometric analysis at 16 weeks

**Parameter**	**Traditional (n=25)**	**Compressive (n=25)**	**p-value**
Bone-to-implant contact (%)	52.3±9.1	68.4±8.7	<0.001*
Bone area fraction occupancy (%)	48.9±8.2	61.2±7.4	<0.001*
Marrow space (%)	51.1±8.2	38.8±7.4	<0.001*
*Statistically significant.
Compressive implants showed 30.8%
higher BIC and 25.2% higher bone density.

**Table 3:** Implant stability quotient (ISQ) over time

**Time point**	**Traditional**	**Compressive**	**p-value**
Placement	71.2±5.4	78.9±4.1	<0.001*
2 weeks	69.8±5.9	79.4±3.8	<0. 001*
6 weeks	72.1±5.2	80.1±3.5	<0.001*
16 weeks	74.1±4.6	79.8±3.2	<0.001*
*Statistically significant.
No secondary stability dip
occurred in the Compressive group
